# Experiences of striving to maintain daily life among women with osteoporosis

**DOI:** 10.1080/17482631.2019.1647402

**Published:** 2019-07-26

**Authors:** Carina Nilsson, Birgitta Lindberg, Päivi Juuso, Malin Olsson

**Affiliations:** Division of Nursing, Department of Health Sciences, Luleå University of Technology, Luleå, Sweden

**Keywords:** Nursing, osteoporosis, phenomenology reflective lifeworld research, women’s health

## Abstract

In order to describe how women with osteoporosis strive to maintain daily life we interviewed 11 women using a reflective lifeworld approach based on phenomenological analysis. Osteoporosis is a major public health concern in the Western world, and is predominant among women. Our findings indicated that meanings of striving to maintain daily life imply a belief in oneself and one’s own capabilities. The women expressly speak out for themselves as a way of finding reconciliation without giving in to the illness. Women with osteoporosis expect to gain support early in the course of their illness. They require advice on how to manage the disease as well as support for striving to maintain daily living. Therefore, it is crucial that the women not only are given information about the disease. Equally important is to establish continuity in healthcare encounters, and that health care offers support founded in the women’s lived experiences with focus on their capacities.

## Introduction

Osteoporosis is considered a major public health concern in the Western world. Globally, osteoporosis causes more than 8.9 million fractures per year, and in the Western world, the lifetime risk of an osteoporosis-related fracture is estimated to be 30–40%, an amount very close to the rates for heart disease (Sözen, Özışık, & Başaran, 2016). The societal burden of osteoporosis in Sweden was noted to be highly underestimated by Borgström, Sobocki, Ström, and Jönsson (2007), and the annual burden of the disease was estimated to increase additionally in the near future. Osteoporosis is a bone disease that effects the entire body, meaning that bone mass or density decreases. When the bones become less dense, they get fragile, which in turn increases the risk of fractures. People with osteoporosis are often afflicted with low-energy fractures, which means that the bone is fractured due to falling (e.g., traumas that non-osteoporotic bone would resist). Osteoporosis is predominant among women and said to be related to the incidence of menopause, where a weakening of bone structure can also be found.

Women who live with osteoporosis have described the diagnosis as a process spanning a longer period of time. In the process of reaching a diagnosis, women have described feelings of being taken seriously but have also experienced the opposite. When being diagnosed, women tend to feel credible but with a long period of uncertainty in mind. Meeting with a specialist of osteoporosis has also been described as beneficial, but experiences of not being taken seriously still remain from previous healthcare encounters (Hansen, Konradsen, Abrahamsen, & Pedersen, 2014). The fear of falling and sustaining a fracture is a common experience among women with osteoporosis (Hansen et al., 2014), and women’s self- awareness, bodily identity, and integrity are also affected by being ill (Dalsgaard Reventlow, Hvas, & Malterud, 2006; Hallberg, Ek, Toss, & Bachrach-Lindström, 2010). Gold (1995) and Hallberg et al. (2010) highlighted quality of life among women affected by fractures related to osteoporosis and showed an increased risk of anxiety and depression, and an effect on self- image and social roles. More recently, Nielsen, Huniche, Brixen, Sahota, and Masud (2013) highlighted emotional difficulties related to attempting to manage osteoporosis in daily life.

When managing daily life, it is evident that women try to make lifestyle changes and to learn to live with the illness, and Hansen et al. (2014) put forward cognitive processes of understanding and meaning creation as important in relation to the new life circumstances for women diagnosed with osteoporosis. When it comes to women’s health, the experience-based knowledge that lies within women’s narratives of being ill is of significance (Olsson, 2017). Despite this, healthcare encounters tend to be focused mainly on the objective view of the body and the biomedical issues related to the disease (cf. Dalsgaard Reventlow et al., 2006; Malterud, 1999). The objective view of the female body has also had, and still has, an impact on how women who seek health care are both met and treated (Connolly, 2001; Malterud, 1999). Werner and Malterud (2003) clearly demonstrated how women’s experiences of patient accounts are strongly driven by behaving as “a credible patient.”

In summary, the literature review indicates that healthcare encounters for women with osteoporosis have a mainly objective view of the female body. In order to understand how women with osteoporosis strive to maintain daily life, it is necessary to add knowledge about women’s strengths concerning coping with an objectified view of the body as weakened. The need for studies that focus on how women with osteoporosis-related fractures maintain their independence in daily living was highlighted by Hallberg et al. (2010). Hansen, Abrahamsen, Konradsen, and Pedersen (2017) show that there is a clear need for developing individual health-oriented support for women who struggle to live with osteoporosis in daily life.

Thus, the purpose of this study is to describe how women with osteoporosis strive to maintain daily life.

## Method

In this study, we have chosen a phenomenological approach. Our approach has the epistemological character of lifeworld theoretical assumptions (cf. Dahlberg, Dahlberg, & Nyström, 2008) when understanding how women with osteoporosis strive to maintain their daily lives. Our goal is to put forward knowledge that is useful in nursing when the goal is to act on lived needs and capabilities expressed by women with osteoporosis. We adopted a reflective stance in the search for meaning, and our ambition was to reflect on what is commonly taken for granted in understanding the strive in maintaining daily life among women with osteoporosis.

### Participants and procedures

In the present study, 11 women diagnosed with osteoporosis participated. The criteria for participation were that their daily lives had been influenced by the illness, and that they were willing to share their experiences. All the women who participated had undergone dual- energy X-ray Absorptiometry (DXA). Their ages ranged from 58 to 78, and as a group, they had experienced symptoms for 2–25 years; 5 women were married, 3 were single, and 3 were widowed; 10 were receiving a state pension, and 1 was unemployed. The women were contacted by means of a purposive chain referral, and the patient association contacted the women about the study. All of those contacted were interested in participating. Information letters were sent to the women to invite them to participate, to give information, and to obtain informed consent. The information letter included a reply form, and after they had agreed to further contact, the authors telephoned each woman to arrange an interview.

### Lifeworld interviews

Individual audio-taped interviews were conducted using a reflective lifeworld approach (RLR) (cf. Dahlberg et al., 2008). To open the interviews and to direct the women’s intentionality we asked the women to tell us about their experiences of striving to maintain their daily life related to living with osteoporosis. As interviewers, we strived to take on a stance of immediacy and presence when interviewing. An interview guide was used with an opening question “Please tell me about your experiences of living with osteoporosis”. Examples of areas concerned during the interviews were; experiences how the illness influenced their lives, challenges in daily living, and their strive to handle daily living. We engagingly used probing questions such as “What is it like?”, “How did you feel?”, “Please, can you tell me more?”, and “Could you exemplify?” to clarify the women’s narrated experiences of the phenomenon. The women were encouraged to reflect on the phenomenon of striving to maintain daily life related to living with osteoporosis during the interviews, and we used responding, directing, and follow-up questions to maintain initiative during the interviews. All the women preferred to be interviewed in their own homes, and this gave the situation an atmosphere of being in the present. The interviews lasted between 40 and 60 min and were transcribed verbatim. Data collection was performed during 2014. The women’s descriptions of the phenomenon varied, even as the boundaries of the descriptions of the phenomenon were evident.

### Data analysis by reflective lifeworld research

In this study, we set out to analyse and describe the phenomenon of managing the challenges of everyday life as described by women with osteoporosis. In our analysis and description, we put forward a structure of essential meanings that explicates the phenomenon under study. The focus of our attention was set to approach lifeworld descriptions that were based in women’s everyday experiences of their worlds (cf. Dahlberg, 2006). The RLR analysis has a tripartite structure with a movement between the whole to the parts and then back to the whole. This movement is significant in analysing meanings of a phenomenon, where meanings are already existing matters seen as bound to subject and context (Dahlberg et al., 2008). In this type of analysis, the knowledge of the whole generates questions about the parts, and knowledge about the parts generates new questions about the whole (Bengtsson, 1991; Dahlberg et al., 2008).

We began the analysis by listening to the audio-taped interviews, and then read and reread the transcribed interviews to gain insight into the text as a whole. After gaining an understanding of the text as a whole, we focused our reading on exploring the phenomenon and searching for meanings of the text in its parts. Meanings of the texts were then related to each other by similarities, as a structure of meaning was explored by clustering meaning-carrying parts and several clusters could be unified by meaning. In this process, we abstracted the meanings in order to reach an essence formation from meanings related to each other and constituting the phenomenon. We viewed the material as a whole, and patterns of meanings were described by the formation of essence.

During the analysis, we explored patterns of meanings from the interview text, and an invariant essence of the phenomenon was explicated. The essence was evident in all clusters and in all of the narrations that were analysed. In the essence, we synthetized the phenomenon’s structure of meaning, and used citations for contextualization and concretization. Dahlberg et al. (2008) state that exploring meanings in data demands a phenomenological attitude in which the researcher’s preunderstanding is bridled; this implies a scrutinizing stance in relation to the meanings of the studied phenomenon, and reflecting on the whole as meanings come to light is a necessity. In this study, our presupposed approach to the phenomenon was bridled in favor of letting it show itself and we constantly questioned our own judgments of general considered certainties.

### Ethical considerations

Information about the study was given, both written and verbally. Participants also had an opportunity to get more information before making decision to participate. Informed consent was obtained both written and verbally. The women were insured confidentially, and that the findings will be presented in a way that no participant would be identified (cf. Polit & Beck, 2016). According to existing Swedish law on ethical review of human research, no ethical review was required. Ethical guidelines and rules were carefully considered to prevent and avoid risks and harms.

## Findings

The essence of meanings of striving to maintain daily life as experienced by women with osteoporosis was understood to encompass an altered life situation where there is a need for support at the same time that one wants to express oneself and one’s own capacities and strengths. When it is possible to find new ways to be engaged and to replace former interests, it is also possible to find meaning in daily living. Meanings of striving to maintain daily life imply a belief in oneself and one’s own capabilities. To expressly speak up for oneself is understood as finding reconciliation without giving in to the illness. We present the findings in four constituents: Resisting the illness by not letting it dominate one’s life; Sheltering one’s independence while needing support; Striving towards feeling well; and Reconciling with the circumstances of life.

### Resisting the illness by not letting it dominate one’s life

Striving to maintain daily life for women with osteoporosis means experiencing a feeling of resisting the illness and not letting it dominate their lives. The women described adapting to a life characterized by pain and bodily limitations, and it was said that this implied realizing their own limitations. At the same time, the women expressed that they had to challenge themselves. The challenge described meant a struggle to get accustomed to an altered daily life.

It doesn’t feel good since I really like to be in movement and so on … but I realize that I cannot do it in the same manner as before.

Women with osteoporosis described how they were constantly aware of the consequences of overly intense exertion. They had to adapt their activities to align with their strength and physical limitations. They also described being afraid of hurting themselves, and this was said to be an important factor in regard to giving up former activities. When letting go of former activities, the women said that they replaced them with other interests, as it was important for them to have something meaningful to do. Women with osteoporosis described being more careful and attentive to their body’s signals because they wanted to avoid pain. The need for exercise was described as a way to resist the illness and to resist physical decline. They said they felt it was important to make time to rest and to always keep in mind doing only as much as they could. The women also described the importance of planning and always being one step ahead. Having routines and exerting control in their daily lives was described as significant. Striving to maintain daily life was said to imply a feeling of having to overcome the illness and resist in order not to just sit down and accept it.

Finally, you think, damn … I have to move on … you have to take the power over the illness … it is not this illness that is gonna control me … as long as possible anyhow.

### Sheltering one’s independence while needing support

When the women talked about striving to maintain daily life they pointed out the importance of having support from others without losing their independence in daily life. To be in an understanding and considerate environment implied support and resulted in decreased anxiety for them. Support meant being encouraged and motivated. The women said that they had to be headstrong in order to safeguard their independence in daily life. An overprotective attitude from others was described as annoying, and a coddling attitude made them feel less autonomous and influenced their self-determination in a negative way. The women emphasized the importance of believing in oneself and of stressing one’s own capacity despite having osteoporosis. They explained to others explicitly when they did not have the strength to take part in various activities of daily life. When others showed a lack of understanding, the women found it important to stress their needs and to speak up.

You know … I’ve shrunk 10 centimeters … I had a small cooling bag [to store in the airplane shelf], and I asked the flight attendant if she could assist me, but she replied that they no longer assist with that. Then I said, you know the bag weighs nothing … you know, I can give tit-for-tat … but what about others who don’t do that?

Women with osteoporosis described that the lack of continuity in health care affected their independence. It was said that if they had been offered advice and support earlier in their illness instead of too late, this would have facilitated their ways of coping. They described that participating in a support group implied acquiring strategies for maintaining daily life and prevented feelings of loneliness and isolation.

The Osteoporosis Association has meant a great deal; we are discussing and planning a lot … meeting others [with the same experience] is strengthening because you can listen to their experiences and what they do to make it a little easier for themselves.

### Striving towards feeling well

The experience of striving to maintain daily life entails a notion of striving for physical and emotional balance in which a feeling of well-being exists. The women with osteoporosis described how they tried to find stimulation for both body and mind by shifting between rest and activity. To be active and engaged was said to be one way to find relief from the illness.

Art history courses, they have been stimulating to attend, and then to exercise, to take walks … and recreational life and such things, it is also really stimulating and gives you an opportunity to rest from the illness.

They described how they could experience distraction from the illness by being outdoors and enjoying recreation. Being able to find meaningful things to do, such as moving around outdoors and enjoying the different smells in the woods and the quiet was restful, as was taking a course or spending time with people who gave them joy. The women described that having fun with friends was important for maintaining daily life, and they strived to focus on doing the things that gave them pleasure. Striving to maintain daily life meant taking care of oneself and feeling safe.

It means a lot to go out, I have friends and I like socializing. We meet each other, my best friends and me, for a dinner during weekends, not every weekend, but often. We watch TV and we are interested in song and music and dance and things like that, and this is really satisfying for me to be together and talk about everything.

### Reconciling with the circumstances of life

Striving to maintain daily life for women with osteoporosis entails a notion of reconciling with the circumstances of life. The women described finding reasons for how their lives had turned out. Engaging in patient associations was said to be one way to improve the conditions for people who were affected by osteoporosis. The women described how they searched for knowledge about the illness, and they felt a need to know what to expect and how they should handle their illness in daily life. They read everything about the illness but still wanted to know more about what others with osteoporosis do to manage their illness.

You have to be a pharmacist in order to understand this treatment. I had read all about osteoporosis and the treatment and the prognosis and yes, everything. And I had to do all this by myself.

Women with osteoporosis described how they strived to never feel sorry for themselves, and they viewed this as one way to avoid falling into a passive state. They described thinking that they had not been struck by a worse illness and tried to feel grateful for that. The women said that they felt hopeful about getting the right medical treatment, and despite not being cured, they hoped that further decline in bone mass could be prevented. Taking responsibility for oneself was said to be important for maintaining life. They described working with their situation in the present instead of thinking about how the future would turn out and whether it would entail pain or immobility.

I believe it has a lot to do with myself … and I cannot expect others to solve things for me … you should have support and help. I believe I have that from my doctor, physiotherapist, family and so … but then you need to work with yourself.

## Discussion

In this article, we suggest that meanings of striving to maintain daily life as experienced by women with osteoporosis, was understood to encompass an altered life situation where there is a need for support at the same time as wanting to express oneself and one’s own capacities and strengths. Striving to maintain daily living meant that the women found new ways to be engaged and to experience meaning in daily living. Meanings of striving to maintain daily life for women with osteoporosis imply a belief in oneself and one’s own capabilities and not giving in to the illness. At the same time the women expressed anxiety due to lack of continuity in care and this effected their strive to maintain daily living. We present the analysis in four constituents: Resisting the illness by not letting it dominate one’s life; Sheltering one’s independence while needing support; Striving towards feeling well; and Reconciling with the circumstances of life. Svenaeus (2001) describe, from a phenomenological point of view, how the structures of human existence always imply an act of balancing between being at home or not being at home in the world. This phenomenon becomes even clearer when people are faced with an illness that affects their everyday life. In this study, we understand that, for women with osteoporosis, the familiar attunement in everyday life is interrupted by pain and physical limitations. When the homelike or balanced attunement is challenged by illness, striving to find a way back to balance is our familiar way of being connected to the world (Svenaeus, 2001). For women with osteoporosis, we have understood that there is a striving in order to become accustomed to their lives being altered.

For women with osteoporosis, the illness demanded adjustments in order to manage both pain and physical limitations. Our findings show that women with osteoporosis had to adapt their activities to align with their strength and what they were able to do. These findings are similar to the results of Hammond and Hirst-Winthrop (2016), who described adjustment as a process of adaptation. People living with chronic illness negotiate a period of change and adaptation; thereafter, they can reach the best possible level of unobstructed daily living. However, some may adjust only partially and have to live with difficulties of an emotional and/or functional nature. The women with osteoporosis stressed how they refused to give in to the illness. This can be understood as the women resisting their illness by making life changes to protect themselves from further harm. In alignment with Svenaeus (2001), this can be understood as reclaiming the familiar sense of harmony that implies a feeling of being at home in everyday life.

In this study, we understand that the women with osteoporosis were attempting to shelter their independence in the realm of needing support from others. The women wanted to keep their independence in daily life, and it was important for them to believe in themselves and to be aware of their own capacity. Kralik, Koch, Price, and Howard (2004) described that identifying and learning how to facilitate control in daily life meant that a sense of independence could be achieved, which entailed taking control of daily living. For people living with long-term illnesses, conscious effort and creativity have been described as required. This conscious effort and creativity have been described as entailing a striving to participate in life, to connect with others, and to develop strategies, as well as finding meaning in life (Whittemore & Dixon, 2008). In our study, a conscious effort among the women with osteoporosis was evident, and they strived for an awareness about their own capacity and belief in themselves.

In this study, our understanding is that sheltering one’s independence when living with osteoporosis requires strong confidence in oneself and solid self-esteem for creating order in daily life. The women with osteoporosis described how the lack of continuity in health care affected their independence. To receive advice at the right time instead of getting support “too late” was said to have a positive effect on the management of their daily life as well as their future. Tarrent, Windridge, Baker, Freeman, and Boulton (2015) describe the gap or lack of continuity in care as a common phenomenon that implies experiences of being rejected and distressed. Additionally, Gulliford, Naithani, and Myfanwy (2006) stressed that continuity in care as crucial if the goal is to focus on patients own views, and thereby secure quality and patient satisfaction, and this is a result that corresponds with the findings of our study on how women with osteoporosis describe their needs. Hansen et al. (2014) showed that women with osteoporosis have experiences of being considered credible as well as non-credible in healthcare encounters. Women who seek health care make attempts to fit in with normative and biomedical expectations. A lot of hard work has been shown to be involved with making symptoms socially visible and physical when consulting healthcare professionals. There is said to be a struggle for credibility and a balance between appearing too weak or too strong or too smart or too unbalanced (Werner & Malterud, 2003). As we understand it, the women expressed a lack of support that can be understood as contributing to feelings of uncertainty and anxiety. The need to receive support at the “right time” can be understood in relation to Lundman and Jansson (2007), who described how living with a long-term illness often implies a need to re-evaluate daily life and find new solutions to manage difficulties. In order to create and maintain a sense of order in life, it is crucial to experience support in managing the course of the disease. Therefore, it seems important that women with osteoporosis experience support characterized by continuity.

Further, our study shows that the required support did not focus exclusively on knowledge about the disease itself. Instead, the women described how they needed support in the process of learning to live with osteoporosis. Connolly (2001) has paid attention to the female embodiment in clinical practice and discussed how different behavior is expected from women than from men, and that in clinical practice, the same behavior is understood very differently depending on the gender of the patient. For women with osteoporosis, this corresponds to the findings of Dalsgaard Reventlow et al. (2006), who showed how results from medical diagnoses often tend to disempower women and leave them with uncertainty and unanswered questions in regard to lack of support about how to reformulate daily living due to illness. For women in this study, it was evident that the lack of advice, lack of support, and lack of continuity in health care negatively affected their ability to manage daily life. Despite the lack of continuity in care, the women with osteoporosis were determined to move forward. Kralik, Price, and Telford (2010) described how, in order to move forward with a sense of the future, people with long-term illnesses undergo a process that means adapting to the illness by learning about oneself and ways to live well with the illness. Therefore, it seems important that women diagnosed with osteoporosis gain support focused on learning to live with the illness upon receiving this diagnosis. Kralik et al. (2010) have described how support focused on self-management can be helpful in order to reclaim a sense of order in daily life. As the findings of our study indicate, women with osteoporosis were in need of re- establishing control in their daily lives and learning to live well with the illness, and it seems like gaining control is crucial for women with osteoporosis when striving to move forward with a positive feeling about the future (cf. Kralik et al., 2010).

The findings of this study show that in order to maintain daily life women with osteoporosis strived for physical and emotional balance, which gave them a sense of well-being and reconciliation with the changed circumstances of their lives. Dahlberg, Todres, and Galvin (2009) showed that well-being is an experience characterized by vitality, including the possibility of movement but also a possibility of peace. These are not contradictory to each other but may rather be prerequisites for each other. In this study, these findings can be understood as the women’s descriptions of being active and engaged, which gave them respite from their illness. According to Gadamer (2004), health manifests itself through overall feelings of well-being, which are possible to achieve despite illness, by being able to enjoy one’s previous roles in everyday life. For women with osteoporosis, this is manifested through their quest to do meaningful things for themselves and to find new ways to participate in previous activities. From this, it can be understood that the women had inner strength that, according to Boman, Häggblom, Lundman, Nygren, and Santamaki Fischer (2015), is a resource for health. To be creative and to find new ways to take part in previous activities implies inner strength and an openness to what life gives one, which is vital for feelings of well-being (cf. Dahlberg et al., 2009). Simultaneously, women with osteoporosis found relief and relaxation through activities with friends. Others have an important role in ill persons’ lives, and Galvin and Todres (2011) stated that belonging and kinship can imply feelings of well-being.

## Methodological considerations

In this study, we recruited the participants from a patient association, and in doing so, there is always a risk of biased sampling. This kind of sample selection often encourages a certain type of informant, which could affect the possibility of transferability. On the contrary, by turning to women who were willing to describe their experiences about maintaining daily life and adjusting their lives to manage the illness, we gained access to meaningful and varying expressions from the women’s points of view. The interviews provided us with rich descriptions of the phenomenon under study, which is known to be crucial in gaining theoretical richness and accuracy. During the interviews, the women could freely express their experiences, and the interviewers used probing interview questions, which was crucial to bridle the presupposed approach to the world in favor of questioning judgments of a certainty commonly considered as general (cf. Dahlberg et al., 2008). By using probing questions, we could steer the interviews to be phenomenon-focused, and the probing also led the women to reflect and express further layers of meaning. Dahlberg et al. (2008) describe that essence is always understood related to its horizons, and this is the approach that we have used when analyzing and attempting to understand the phenomenon under study. In our analysis, we have strived for a bridled approach related to the figure and background of the situation of maintaining daily life when living with osteoporosis.

## Conclusion

In conclusion, this study shows that meanings of striving to maintain daily life as experienced by women with osteoporosis, was understood to encompass an altered life situation where there is a need for support at the same time as wanting to express oneself and one’s capacities and strengths. Striving to maintain daily life meant that the women found new ways to be engaged and experience meaning in daily life. Meanings of striving to maintain daily life imply a belief in oneself and one’s capabilities and not giving in to the illness. In order to support women with osteoporosis, it is crucial to establish continuity in healthcare encounters as well as to support women’s strive to learning to live with the illness. This can be realized by attending to the women’s needs of being offered support at the time of diagnosis, and not singularly being given information about the disease. Additionally, there is an evident need for an altered focus towards the women’s lived experiences when trying to manage the illness and live well.
